# Leveraging the Expertise of the New Jersey Mosquito Control Community to Jump Start Standardized Tick Surveillance

**DOI:** 10.3390/insects10080219

**Published:** 2019-07-24

**Authors:** Andrea M. Egizi, James L. Occi, Dana C. Price, Dina M. Fonseca

**Affiliations:** 1 Tick-Borne Disease Laboratory, Monmouth County Mosquito Control Division, Tinton Falls, NJ 07724, USA; 2 Center for Vector Biology, Department of Entomology, Rutgers University, New Brunswick, NJ 08901, USA; 3 Public Health Environmental and Agricultural Laboratory, New Jersey Department of Health, Ewing, NJ 08628, USA; 4 Department of Plant Biology, Rutgers University, New Brunswick, NJ 08901, USA

**Keywords:** integrated pest management, vector-borne diseases, vector surveillance, citizen science, American dog tick

## Abstract

Despite the rising incidence of tick-borne diseases (TBD) in the northeastern United States (US), information and expertise needed to assess risk, inform the public and respond proactively is highly variable across states. Standardized and well-designed tick surveillance by trained personnel can facilitate the development of useful risk maps and help target resources, but requires nontrivial start-up costs. To address this challenge, we tested whether existing personnel in New Jersey’s 21 county mosquito control agencies could be trained and interested to participate in a one-day collection of American dog ticks (*Dermacentor variabilis*), a presumably widespread species never before surveyed in this state. A workshop was held offering training in basic tick biology, identification, and standard operating procedures (SOPs) for surveillance, followed by a one-day simultaneous collection of *D. variabilis* across the state (the “NJ Tick Blitz”). In total, 498 *D. variabilis* were collected from 21 counties and follow-up participant surveys demonstrated an increase in knowledge and interest in ticks: 41.7% of respondents reported collecting ticks outside the Tick Blitz. We hope that the success of this initiative may provide a template for researchers and officials in other states with tick-borne disease concerns to obtain baseline tick surveillance data by training and partnering with existing personnel.

## 1. Introduction

The northeastern United States currently have the largest burden of tick-borne diseases (TBDs) in the nation, due primarily to the concentration of Lyme disease within the region (~81% of 38,069 Lyme disease cases in the United States (US) in 2015 [1]) but also increasing prevalence of anaplasmosis, babesiosis, and spotted fever rickettsioses [2,3]. Ticks are both a threat to human health and to economic health: According to a recent estimate, healthcare costs associated with diagnosis and treatment of Lyme disease could total as much as $1.3 billion per year [4] and tick-borne illnesses are a common drain on the labor force especially to those spending time outdoors, such as agricultural workers [5,6]. 

The number of tick-borne disease cases in the US have increased annually since ca. 2000 and new pathogens are continually emerging; in fact 40% of all known tick-borne pathogens were described in just the last 20 years [7,8]. The tick-borne disease landscape in the northeastern US has therefore undergone dramatic shifts since the emergence of Lyme disease in the 1980’s including the northward expansion of lone star ticks (*Amblyomma americanum*) and associated pathogens [9] as well as growing recognition of human infections with deer tick virus (DTV), a new lineage of Powassan virus vectored by *Ixodes scapularis* [10]. Furthermore, questions about the changing epidemiology of spotted fever rickettsioses in the US [11] are especially pertinent in the northeast, where human cases are increasing but the causative agent, *Rickettsia rickettsii*, is rare in the presumed vector, American dog ticks (*Dermacentor variabilis*) [12]. The southern Gulf coast tick, *A. maculatum*, is a vector of related pathogen *R. parkeri* and has recently expanded into Maryland and Delaware [13] but its penetration further north is unknown. As TBD incidence has been linked to climate, the situation is expected to worsen [14].

Against the backdrop of a high TBD burden, integrated tick and tick-borne disease management strategies in the Northeast region are broadly missing [15]. The first step to devising strategies to minimize disease burden is to assess which tick species are present, their abundance and their phenology [16,17]. Unfortunately, in much of the northeast there is currently very little funding/infrastructure available to conduct even basic tick surveillance. In particular, while some university, county or state programs test for pathogens in ticks submitted by residents and physicians (passive surveillance) this practice is discouraged by the US Centers for Disease Control and Prevention (CDC) in favor of active tick and tick-borne pathogen surveillance similar to existing programs that track mosquitoes and mosquito-borne pathogens (https://www.cdc.gov/lyme/removal/index.html).

The lack of information on ticks and tick-borne pathogens means that we are unlikely to notice changes until prevention is no longer feasible, i.e., after infestations have established or human disease cases have become common. Importantly, due to the potential for wide cross-reactivity among closely-related and/or emerging pathogens in standard serological testing, relying on human case reports as a proxy for pathogen/tick surveillance is suboptimal. For example, extensive cross-reactivity among *Rickettsia* bacteria in diagnostic testing of humans obscures large differences in pathogenicity [18,19]: The human disease-based surveillance cannot distinguish between potentially fatal *R. rickettsii*, the agent of Rocky Mountain Spotted Fever (thought to be transmitted by *D. variabilis* in the Eastern US), moderately pathogenic *R. parkeri* (transmitted by *A. maculatum*) or even apparently non-pathogenic *R. amblyommatis*, a common (>25% infection rates, [20]) bacterium found in the lone star tick, *A. americanum.* These three agents have widely varying degrees of pathogenicity and occur in three different tick species (all of which commonly parasitize humans, [21]) with overlapping distributions in the eastern United States, underscoring the epidemiological need for entomological surveillance. 

Tick-borne disease surveillance and education in the northeast has centered on Lyme disease (LD) since the early 1980’s when this emerging disease was first linked to the spirochete *Borrelia burgdorferi* found in ticks [22,23,24]. Dozens of studies have mapped LD cases and the distribution of its vector, the blacklegged or deer tick (*I. scapularis*) in a variety of northeast and north-central states (reviewed by [25], and updated by [26]). In those studies, ticks primarily came from surveys on deer associated with deer-check stations (30.1%), followed by public submissions (18.1%), while flagging/dragging made up just 7.5% of collections [25]. Many of these surveys were able to document significant changes in *I. scapularis* populations, such as local increases in abundance or geographic expansions into new areas [27,28,29]. As awareness of other TBDs began to increase in the northeast including anaplasmosis and babesiosis [30,31], new surveys began tracking the distribution of their causative agents (*Anaplasma phagocytophilum* and *Babesia microti*, respectively). However, because these pathogens are also vectored by *I. scapularis*, the singular focus on this species continued (e.g., [32,33,34], among many others). In fact, only a few studies within the northeast have specifically targeted other species such as *A. americanum* [35,36,37] and *D. variabilis* [38,39]. To the best of our knowledge, in many northeastern US states (including New Jersey) there has never been a systematic survey of *D. variabilis* or of the pathogens it may carry, as this tick species favors open fields such as grassy roadsides and meadows instead of the forests where *I. scapularis* thrives [40]. This is particularly a concern as some areas are seeing increasing numbers of encounters between humans and *D. variabilis* [41]. 

Concurrent with the early focus on LD in tick surveys, surveys examining public knowledge about ticks and evaluating the success of prevention education in the northeast US also focused on *I. scapularis*. While most studies have found that public awareness of LD is high, the use of personal precautions is consistently low [42,43]. Overall, the public knows very little about tick-borne diseases other than LD [44,45].

**Figure 1:**
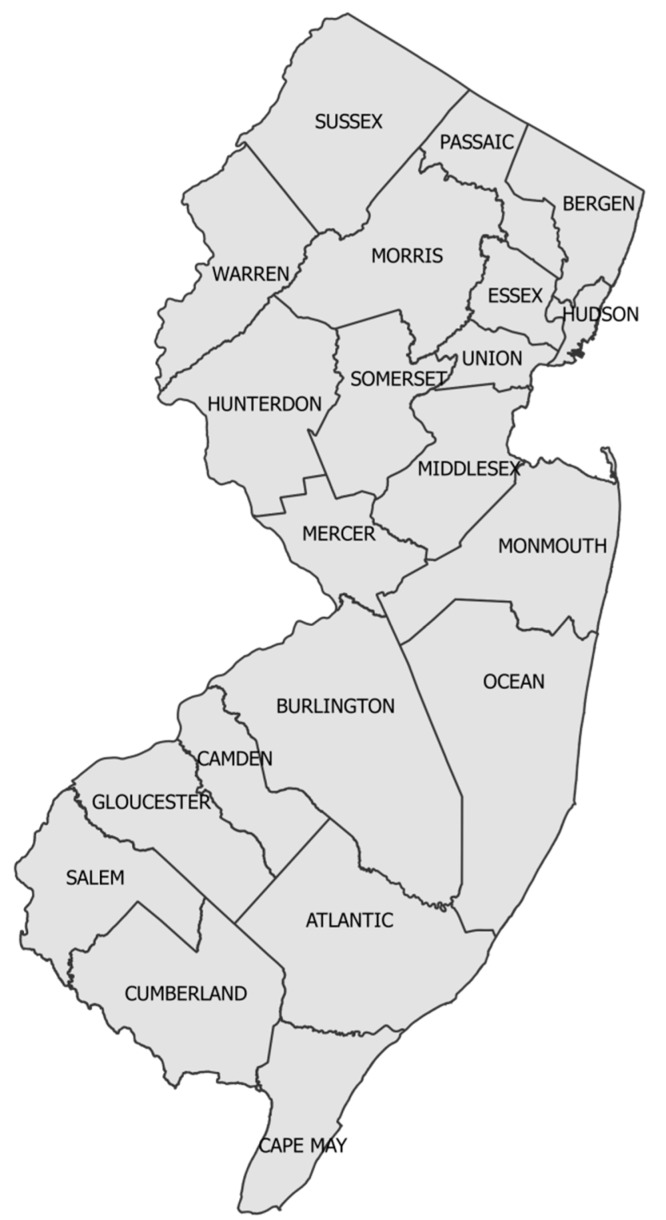
Map of New Jersey with 21 counties, each of which has a locally funded mosquito control program.

The primary objective of our study was to assess the interest and proficiency of existing agencies in New Jersey (NJ) dedicated to pest management and public health to provide state-wide standardized tick surveillance. We targeted the NJ mosquito control community that has agencies in all 21 NJ counties (Figure 1), two professional organizations (the New Jersey Mosquito Control Association (NJMCA) and Associated Executives of Mosquito Control Work in NJ, Inc.) and a long history of science-based mosquito control research and practice [46]. A secondary objective was to obtain the first NJ statewide snapshot of the putative *Rickettsia* vector *Dermacentor variabilis*. We present our experience, results and lessons learned from trialing a “Tick Blitz” approach with this community, supported by a one-day workshop, where surveillance SOPs (standard operating procedures) and supplies were provided. Our aim was to evaluate if a Tick Blitz-like approach could act as a crucial first step towards developing a quorum of skilled personnel and statewide interest conducive to investment in a larger tick-surveillance program.

## 2. Materials and Methods

### 2.1. Recruitment and Training

Mosquito control agencies were recruited via an in-person announcement at a monthly meeting of the Associated Executives of Mosquito Control of New Jersey (“Associate Execs”) in the fall of 2017 requesting letters of support for a Northeast IPM Partnership Grant application. We received letters from 15 out of 21 counties plus the NJ State Office of Mosquito Control Coordination (OMCC; housed in the New Jersey Department of Environmental Protection). After the grant was awarded, we made a second announcement at one of the Associate Execs monthly meetings and sent a follow-up email to the organization’s list-serve with additional details asking mosquito control professionals to sign up for a workshop that was held on 4 May 2018 and participate in a one-day “NJ Tick Blitz” that was held on 10 May 2018. 

50 attendees from 24 agencies (20/21 county mosquito control agencies plus the OMCC, New Jersey Department of Health, Rutgers University and US Department of Agriculture—Animal and Plant Health Inspection Service) attended the training. At the workshop, speakers from Rutgers University and the Monmouth County Mosquito Control Division, Tick-borne Diseases Lab provided information about tick-borne pathogens and tick biology, identification and environmental collecting (including a hands-on demonstration). Detailed information about the Tick Blitz including site selection and additional information regarding surveillance for *D. variabilis*, the focal species, as well as surveillance supplies (below) were also provided. 

### 2.2. Site Selection

In contrast to forest dwelling ticks such as *I. scapularis*, *D. variabilis* typically occupies old field habitats and ecotones adjacent to meadows [47,48]. Each participating county mosquito control agency was given a document with pictures and examples of *D. variabilis* habitat and they were instructed to choose at least two sites within their county that matched the description: One primary and one backup. Pictures and GPS coordinates of these sites were sent to Tick Blitz organizers to review and assess habitat suitability for *D. variabilis*. The organizers reviewed the landscape at each site using Google satellite maps and occasionally Google street view, and gave each agency feedback on whether the site would be suitable and which areas within the site would be ideal for sampling. 

### 2.3. Tick Surveillance 

**Figure 2:**
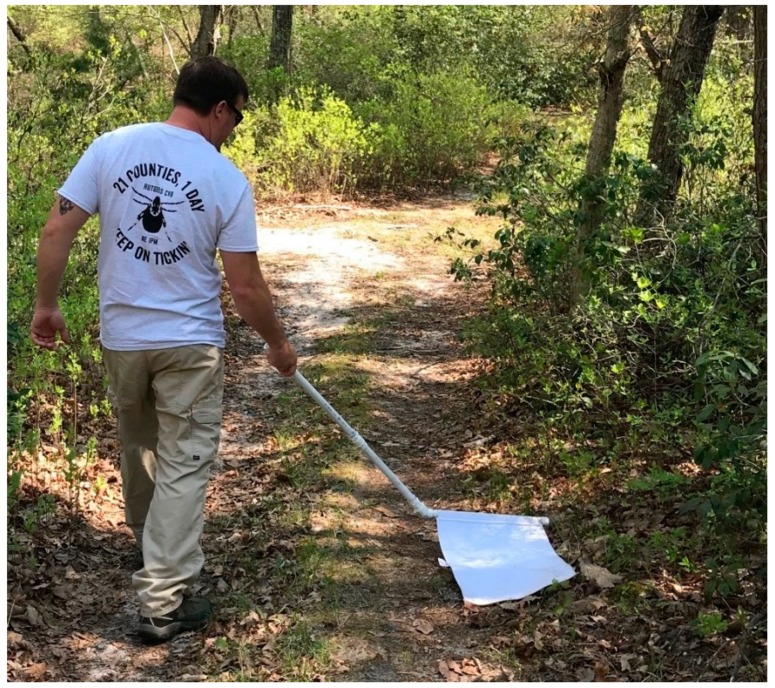
New Jersey (NJ) Tick Blitz participant using provided tick sweep. Original design by [49] with modifications by Benedict Pagac and James Butler. Photo courtesy Jonathan Cassidy and Joe New, Burlington County, NJ.

Emphasis was placed on use of a standardized tick collection protocol at all sites. To facilitate this, tick collection kits were provided to each participating county mosquito control agency. Each kit placed inside a cloth drawstring bag contained: A collapsible “tick sweep” (modified from [49] by Benedict Pagac and James Butler, Army Public Health Command—Atlantic), a NJ Tick-Blitz t-shirt, a roll of masking tape, a box of ziploc bags, a permanent marker and sample collection sheets. The sample collection sheets instructed participants to record the date and time of the collection, collector’s name, county, site number, and transect number; there was also a blank space for additional notes (e.g., weather conditions or issues encountered). We also provided a cardboard box for courier pickup: A courier service was hired to visit each county, pick up collected ticks and transport them to the Rutgers Center for Vector Biology (CVB) for processing. Tick sweeps (a sampling device with a long, bent handle allowing the cloth to contact the ground, [49]) were chosen both due to the type of habitat being targeted for American dog tick sampling (i.e., edge habitat between wooded areas and open grass) and because they were relatively easy to mass-produce. In contrast to Carroll and Schmidtmann [49], who used the device to sweep back and forth in front of the investigator’s path, we instructed participants to walk with the sweep at their side, allowing them to sample the taller grass/ecotone more likely to contain ticks while staying in shorter grass or along trails (Figure 2) thereby reducing their exposure to ticks. Thirty sweeps were manufactured using polyvinyl chloride (PVC) pipe and crib flannel (Buy Buy Baby, cat#14814620, Union Township, NJ, USA). The 0.25 m^2^ flannel was folded around the pipe and sewn to allow easy removal for washing (see Figure 2).

Participants were instructed to measure out 300 m transects along edge habitat at sites selected earlier (one transect per site). Each transect was sampled with the tick sweep held to the side at a slow, steady pace and participants were told to stop every 20–30 m to inspect for ticks. Ticks were removed from the sweeps with masking tape and placed in Ziploc bags with a completed label. This removal methodology was chosen as opposed to forceps and vials to minimize handling time as most participants were first-time tick collectors with job responsibilities outside this project. Recorded length of sampling varied across teams from under 1 h excluding travel time to over 3 h with additional (i.e., more than the 2 requested) sites visited. 

On “Tick Blitz Day,” 50 participants collected ticks in 21 counties. They were instructed to begin collecting simultaneously throughout the state at 10 am Eastern Standard Time (EST). Collected ticks were kept refrigerated until they were picked up by the courier service and brought to the CVB, where they were removed from the tape and identified to species and stage by experienced tick researchers using established keys (e.g., [50]). Due to the recent detection of *Haemaphysalis longicornis* in New Jersey [51] and at the time lack of available keys to distinguish them from native species (but see [52]) ticks in the genus *Haemaphysalis* were identified by DNA sequencing of the barcode locus in the mitochondrial cytochrome c oxidase gene [53]. Very occasionally, non-tick arthropods were picked up along with ticks on the tape, but that bycatch was ignored.

Statewide maps of tick collections were created in QGIS (https://qgis.org/). *D. variabilis* and *A. americanum* were set aside for *Rickettsia* spp. testing [54]. 

### 2.4. Participant Surveys

Pre- and post-tests were administered to participants during the 4 May training. Each paper survey contained five questions (4 multiple choice and 1 open-ended) designed to quickly evaluate the participants’ level of knowledge about ticks and tick-borne diseases before and after the training. Pre- and post-test questions were different but judged to be similar in difficulty by a panel of three researchers. 

After the conclusion of the project in December 2018, a final survey was administered through Qualtrics (Qualtrics, Provo, UT, USA) to examine the participants’ overall experience with the NJ Tick Blitz. This survey consisted of 15 questions on topics such as their knowledge/comfort level with ticks before and after the Tick Blitz, whether or not they had done additional tick collecting outside the Tick Blitz, and what could be improved if the Tick Blitz were repeated. The link was sent by email, participants were given 30 days to respond and responses were collected anonymously. Data was analyzed using the “Data and Analysis” tab in Qualtrics. 

## 3. Results

### 3.1. Tick Surveillance

**Figure 3:**
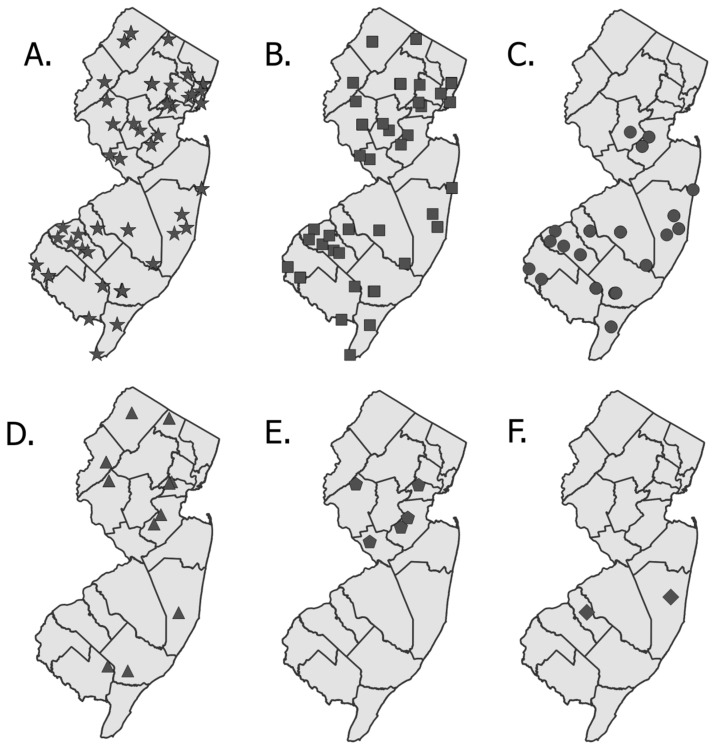
Map of New Jersey plotted with (**A**) all 51 sites sampled for the 2018 Tick Blitz; and (**B**–**F**) Sites where each tick species was collected: (**B**) *Dermacentor variabilis* (Total of 498 ticks); (**C**) *Amblyomma americanum* (238 ticks); (**D**) *Ixodes scapularis* (37 ticks); (**E**) *Haemaphysalis longicornis* (36 ticks); (**F**) *Haemaphysalis leporispalustris* (2 ticks).

Fifty sites in all 21 New Jersey counties were sampled for ticks on the morning of 10 May 2018 between approximately 10 am and 12 pm (Figure 3A). An *a posteriori* evaluation of the site sampled in Essex County, where no ticks were collected, indicated the habitat did not match the guidelines therefore a second site in Essex was sampled on 16 May bringing the total sites sampled to 51. Ultimately, *D. variabilis* ticks (*N* = 498) were collected from all 21 NJ counties (Figure 3B). Other species collected were *A. americanum* (*N* = 238, Figure 3C), *I. scapularis* (*N* = 37, Figure 3D), *H. longicornis* (*N* = 36, Figure 3E), and *H. leporispalustris* (*N* = 2, Figure 3F) (Supplementary Table S1). In general, these incidental collections reflected known distributions of these species in NJ, i.e., a primarily southern distribution of *A. americanum* and a statewide distribution of *I. scapularis*. Specimens of *H. longicornis* were collected from both counties with known populations of this species prior to 10 May 2018 (Hunterdon and Union counties) as well as in two new counties with no prior detections (Middlesex and Mercer). Both specimens of the rabbit tick *H. leporispalustris* were immatures: A larva from Camden County and a nymph from Ocean County.

### 3.2. Participant Surveys: Pre- and Post-Tests

Forty-eight attendees to the Tick Blitz Workshop completed both a pre-and post-test. Most respondents answered the questions correctly (Table 1). The lowest scoring question was pre-test question #1, where many respondents remembered three medically important tick species yet did not realize there are actually more than 10 species in New Jersey (including several that do not bite humans [55]), and post-test question #2, where many accurately remembered that adults have taken a second bloodmeal and thus likelihood of carrying a pathogen is higher, but did not recall from our lecture that nymphs are more likely to transmit a pathogen to humans because they are easier to miss during tick checks (55.3% of respondents answered “adults,” vs. 42.6% “nymphs”) (Table 1). 

One additional question was included on each test but was not scored. On the pre-test, this question asked if participants were familiar with methods to survey ticks, and if so, to give an example. Seventy-five percent of respondents said they were familiar with tick collection methods, and 69.2% of those named tick drags (only 3.8% mentioned CO_2_ traps). On the post-test this question asked “Do you expect surveying for ticks will be much different from surveying mosquitoes?” and 76.6% of respondents selected “Yes.” 

Overall, the post-test mean score (mean ± SD = 3.45 ± 0.68) was higher than the pre-test mean score (2.94 ± 1.12) (Paired *t*-test, *p* = 0.0212).

### 3.3. Participant Surveys: Final Survey

The final survey was sent to participants that both attended the training workshop and participated in Tick Blitz collection (*N* = 45). Responses were received from 25 participants, (55.6% response rate). Results of the survey indicate mosquito control professionals in NJ have ample exposure to ticks during their job and everyday lives (72% encounter daily or frequently) and most were either slightly (36.0%) or moderately (48.0%) knowledgeable about ticks prior to the Tick Blitz (Table 2). 

After the Tick Blitz, knowledge increased slightly, with participants who were slightly knowledgeable before becoming moderately knowledgeable, and participants who were moderately knowledgeable becoming very knowledgeable (χ^2^ = 25.9, df = 6, *p* = 0.0002). After the Tick Blitz, participants felt most comfortable answering residents’ questions (100% either extremely or somewhat comfortable) but least comfortable identifying ticks to genus (75% extremely or somewhat comfortable).

In total, 41.7% of participants were inspired to collect ticks outside of or after the Tick Blitz (Table 2) and 83.4% said they definitely or probably would collect ticks in 2019. Most of those collecting outside the Tick Blitz had a background in Biology (26.7%). Interestingly, participants that collected fewer ticks than they expected during the Tick Blitz were less likely to have collected afterwards (χ^2^ = 7.13, df = 2, *p* = 0.028). 

### 3.4. Surveillance Website

Tick identification results were made available to participants using a surveillance website platform hosted at Rutgers University (http://acari.rutgers.edu/tickblitz/). Each county received a unique login and password that participants could use to enter site information and review data. They could also view statewide data embedded in a Google Map for each tick species (Google Inc., Mountain View, CA, USA). A subset of the data (aggregated by county to obscure sensitive site locations) is available to the public via a link on the site.

## 4. Discussion

The New Jersey Tick Blitz successfully collected specimens of the target species *Dermacentor variabilis* throughout the state of NJ and demonstrated that professionals in local agencies dedicated to pest management and/or public health can be trained and, importantly, are interested, motivated and competent to participate in statewide tick surveillance activities. 

We note that one-day sampling does not give a proper estimate of tick density or abundance at sites, as there may be day-to-day fluctuations in questing tick populations within a site due to weather, host availability and other factors [56,57,58,59,60]. Also, sites chosen to sample within a county may not be representative of tick populations in the county as a whole. Additionally, because there are no standardized leave-on-site traps or other investigator-independent strategies for tick surveillance, having different tick collectors (many relatively inexperienced) is likely to introduce variability in collections across locations, for example each group may walk faster/slower or be more or less likely to spot tiny larvae on the flag, which could affect numbers and/or species of ticks collected.

Despite the limitations discussed above, the New Jersey Tick Blitz was able to improve on our general understanding of tick distributions in New Jersey. Resulting data clearly supported the *a priori* hypothesis [55] that *D. variabilis* is widespread throughout the state. We also learned that the distribution of *A. americanum* extended farther northward than previously thought [37] with specimens collected from Middlesex and Somerset counties. Importantly, first time detections of the exotic tick *H. longicornis* in Mercer and Middlesex counties prompted the US Department of Agriculture and the NJ Department of Agriculture to work with livestock facilities in these counties to protect their animals and spurred additional surveillance efforts for this tick species in NJ. 

The collection of two specimens of *H. leporispalustris* was intriguing as this species is not typically sampled in collections of questing ticks due to their host specificity [61,62]. In fact, earlier collections of questing *Haemaphysalis* in Union County NJ from 2013, originally presumed to be *H. leporispalustris*, were later identified as *H. longicornis* [63]. The presence of both these species in questing tick collections in NJ emphasizes the need for careful identification to distinguish these two species [52]. Of note, despite the ongoing northward expansion of *A. maculatum* into Delaware and Maryland [13], and its utilization of similar types of habitat and seasonal timing as *D. variabilis* [64], we did not detect *A. maculatum* during our sampling in NJ. It is possible *A. maculatum* has not yet made it across the Delaware Bay, or alternatively, populations are still low enough that they could not be detected using our sampling approach. Indeed, there is evidence that capturing and sampling hosts directly may be a more sensitive means to detect nascent tick populations than flagging/dragging [65,66]. 

Both the workshop pre- and post-tests and final survey demonstrated that New Jersey mosquito professionals were already somewhat experienced and knowledgeable about ticks prior to the Tick Blitz, and that the workshop and overall Tick Blitz experience achieved significant improvement in their knowledge and comfort levels. It also captured a noteworthy level of interest and enthusiasm for working on ticks: 41.7% of participants reported collecting ticks outside of the Tick Blitz, despite that task falling outside their job duties. This was especially so for those with a prior background in biology, indicating a strong natural curiosity and intrinsic motivation among this group of professionals. 

However, there were aspects of the Tick Blitz primarily associated with the workshop that can be improved. Open-ended comments from participants included suggestions to break attendees up into smaller groups, giving each person time to handle the tick sweep and try collecting, as well as getting real-time feedback from trainers. There were also suggestions to improve the lecture portion, including providing physical specimens to examine under the microscope for the identification (ID) portion and more advanced (e.g., beyond genus level) ID training. As a result, we recommend that other groups wishing to implement a Tick Blitz in their territory give their participants more hands on experience in both viewing and identifying ticks as well as handling and collecting ticks in the field. Participants were especially eager to have direct feedback from the trainers (“Am I doing this right?”). The receipt of feedback can be an important component of learning [67] and effective feedback has been shown to improve retention of volunteers in activities like citizen science projects [68] and contributions to online data pools [69]. In particular, lack of encouraging feedback may have contributed to participants’ concerns about their competency in collecting ticks (i.e., feelings that they collected fewer ticks than they should have), thus affecting their confidence and motivation to participate in additional sampling. 

As a result, we recommend better managing participants’ expectations so that they understand there are myriad reasons why they may collect few ticks in a given site or on a given day, and that this may not necessarily reflect the tick abundance at that site or their collecting ability. Specific examples from the literature or the instructor’s experience will help improve the trainee’s confidence and prevent them from becoming discouraged with tick collection. Lack of confidence in the results obtained may also help explain why individuals who collected fewer ticks than expected were less likely to collect after the Tick Blitz, although an alternative explanation is that they simply felt there were not many ticks in their county and did not see a need for additional surveillance. In either case, a better understanding of variability in tick collection and the drawbacks of current surveillance is needed [70,71].

In the last part of the survey, we asked the respondents what they thought were the constraints to developing standardized tick surveillance in New Jersey. We found that the majority of participants were already highly motivated from personal experience or that of their employees (72.7%) and resident inquiries (85.0%), however most noted the need for specific funding for tick surveillance (90.0%), training in tick identification (90.9%), and standard operating procedures (SOPs) for tick collection (100.0%) (Table 2). This is an encouraging sign that if funding and better educational support was provided, mosquito control professionals would be willing to enact more formal tick surveillance and their constituents would be supportive. In our experience, building an exploratory tick surveillance program by funding mosquito control professionals is an excellent way to leverage existing resources.

We are confident that the existing significant experience with standardized surveillance practices among the extensive NJ network of mosquito control professionals facilitated training in tick surveillance and the quality of the resulting data. This is an asset that is often missing in other US states [72]. However, the same workshop combined with a hands-on Tick Blitz could be implemented for other types of professionals such as private pest control operators. In fact, in our experience even interested citizens can become effective pest managers if educated and guided [73]. These approaches are not meant to replace a properly funded and well-designed tick surveillance program, where experienced collectors visit a range of sites multiple times over a season. However, in areas lacking such capability or wishing to build capacity, a “Tick Blitz”-like approach could provide critical baseline data on local tick populations that could be used to justify the establishment of a larger program. 

## 5. Conclusions

While we acknowledge that the New Jersey Tick Blitz was conceived as a pilot study with inherent limitations and biases, it nonetheless added significant new knowledge on tick distributions in New Jersey. The mosquito control professionals that participated indicated in their survey responses that the experience provided important training, materials, and risk information, helping them to address the evident and pressing TBD concerns of their residents. On a broader scale, the success of this initiative may provide a template for researchers and government officials in other states with tick-borne disease concerns to obtain baseline tick surveillance data by training and partnering with existing personnel. This data can then be leveraged to secure additional funding for surveillance projects to protect human health by monitoring the changing tick-borne disease landscape.

## Figures and Tables

**Table 1:** Graded responses to pre- and post-tests taken at Tick Blitz workshop on 4 May 2018.

Question No.	Text of Question	Type of Question	% Correct (*N* = 48)
Pre-test 1	“Approximately how many tick species are known to occur in NJ?”	Multiple choice	50
Pre-test 2	“Ticks are active only during the warmer months of the year just like mosquitoes”	True or False	86.4
Pre-test 3	“Like mosquitoes only adult ticks bite”	True or False	97.7
Pre-test 4	“How many different human pathogens are known to be transmitted by ticks in NJ?”	Multiple Choice	86.4
Post-test 1	“Which tick genus can be differentiated from all the others based on the location of the anal groove?”	Multiple Choice	89.4
Post-test 2	“Which tick stage is the most likely to transmit a pathogen to humans?”	Multiple Choice	42.6
Post-test 3	“Where can people be exposed to ticks?”	Checkboxes	77.1
Post-test 4	“Do ticks in NJ transmit any deadly diseases?”	Yes or No	97.9

**Table 2:** Results of Final Survey of Tick Blitz participants, *N* = 25 responses.

Survey Section	Question	Answers	% Respondents
Participant background	Years in mosquito control	0–5	32.0
		6–10	12.0
		11–20	36.0
		21–30	8.0
		More than 30 years	12.0
	Experience outside mosquito control?	Yes	64.0
		No	36.0
	If yes to above, Other fields with experience	Biology	43.8
		Environmental science	25.0
		Parks & Recreation	12.5
		Public Health	31.3
		Public works	0.0
		Other (write-in answers included retail, construction, food service, landscaping, private sector pest management, etc.)	93.8
Pre-Tick Blitz questions	How often participants encountered ticks	On a daily basis	28.0
		Frequently (every couple weeks)	44.0
		Occasionally (a few times a year)	24.0
		Rarely (once or twice in life)	4.0
		Never	0.0
	Level of knowledge about ticks prior to Tick Blitz	Not at all knowledgeable	0.0
		Slightly	36.0
		Moderately	48.0
		Very	4.0
		Extremely knowledgeable	12.0
Tick Blitz experience	How did tick collections compare to expectations?	Fewer than expected	41.7
		About the same as expected	37.5
		More than expected	20.8
	Rating of each aspect: (First number = extremely + very effective, Second number = moderately + slightly effective)	Advertising about the Tick Blitz	87.5, 12.5
		Collection kit provided	95.8, 4.2
		Communication from organizers	91.7, 8.3
		Guidance for site selection	87.5, 12.5
		Hands on portion of workshop	75.0, 25.0
		Incentives to participate	79.2, 20.8
		Lecture portion of workshop	95.8, 4.2
		Standard operating procedures (SOPs) provided	95.8, 4.2
		Website for entering data	87.5, 12.5
	Aspects of Tick Blitz that could be improved	Advertising about the Tick Blitz	4.2
		Collection kit provided	8.3
		Communication from organizers	4.2
		Guidance for site selection	8.3
		Hands on portion of workshop	20.8
		Incentives to participate	12.5
		Lecture portion of workshop	12.5
		SOPs provided	0.0
		Website for entering data	0.0
Post- Tick Blitz questions	Level of knowledge after Tick Blitz	Not at all knowledgeable	0.0
		Slightly	0.0
		Moderately	37.5
		Very	45.8
		Extremely knowledgeable	16.7
	Comfort level: (First number = extremely + somewhat comfortable, second number = neither uncomfortable nor comfortable + somewhat uncomfortable)	Answering residents’ questions about ticks	100.0, 0.0
		Collecting ticks	95.8, 4.2
		Identifying ticks to genus	75.0, 25.0
		Naming tick-borne pathogens in NJ	91.6, 8.4
		Protecting myself from tick bites	100.0, 0.0
		Recognizing tick habitat	91.6, 8.4
	Collected ticks outside of (after) the Tick Blitz?	Yes	41.7
		No	58.3
	If yes, on how many days?	One	30.0
		2–5	20.0
		6–10	20.0
		On a regular basis (weekly, monthly, etc.)	30.0
	Plans to collect ticks next year (2019)?	Definitely yes	41.7
		Probably yes	41.7
		Might or might not	16.7
		Probably no	0.0
		Definitely no	0.0
Tick Surveillance needs in NJ	Which of the following items would your county need to establish a tick surveillance program? (% Has, % Need)	Actionable outcomes (what to do w/info)	40.9, 59.1
		Detailed SOPs for tick collection.	0.0, 100.0
		Employee motivation	72.7, 27.3
		Expertise in Tick ID	9.1, 90.9
		Funding for supplies/equipment	10.0, 90.0
		Funding for personnel	41.2, 58.8
		Guidance from NJ State Office of Mosquito Control Coordination (OMCC)	63.6, 36.4
		Guidance from Rutgers	50.0, 50.0
		Legal authority	47.4, 52.6
		Permission from administration	42.1, 57.9
		Support of residents	85.0, 15.0

## Supplementary Materials

The following are available online at https://www.mdpi.com/2075-4450/10/8/219/s1, Table S1: Tick species and life stages collected during the 2018 New Jersey Tick Blitz, by county.
